# Prevalence of Suspected Cases of Hyperthyroidism in Jeddah by Using Wayne’s Scoring Index

**DOI:** 10.7759/cureus.11538

**Published:** 2020-11-17

**Authors:** Mohammed Qashqary, Mansour Tobaiqy, Manal M Al-Sutari, Alaa Mujallad, Intisar Alsheikh

**Affiliations:** 1 Family Medicine, University of Jeddah, Jeddah, SAU; 2 Family and Community Medicine, University of Jeddah, Jeddah, SAU; 3 Pharmacology, College of Medicine, University of Jeddah, Jeddah, SAU; 4 Nursing, University of Jeddah, Jeddah, SAU

**Keywords:** key words: hyperthyroidism, jeddah, wayne's index

## Abstract

Introduction

Hyperthyroidism is one of the primary dysfunctional thyroid diseases that results from abnormally high thyroid hormone secretion. It leads to metabolic derangement that manifests as heightened vital functions and metabolism and hyperdynamic blood circulation. Hyperthyroidism jeopardizes one’s quality of life and hampers the patient’s job performance. The disease is chronic but diagnosable and treatable and can be kept under control medically. We aimed to estimate the prevalence of suspected hyperthyroidism cases among a population sample in Jeddah city by using Wayne’s scoring and delineation of factors associated with the disease.

Materials and methods

A cross-sectional study design was used. A total of 346 participants were conveniently selected from two shopping malls in Jeddah city during a five-day health awareness campaign. The data were collected by certified physicians, according to Wayne’s score inventory.

Results

The study included 194 women (56.1%) and 152 men (43.9%). The participants’ ages ranged from 16 to 56 years. The Wayne’s scores showed 80.6% were euthyroid, 18.2% equivocal, and 1.2% have hyperthyroidism. Bronchial asthma was significantly associated with hyperthyroidism in men, while obesity was significantly associated with hyperthyroidism in women. Age, gender, level of education, and family history of chronic diseases, including thyroid disorders, were not significantly associated with hyperthyroidism.

Conclusions

Wayne’s scoring for hyperthyroidism, which is a noninvasive tool, in addition to its detection of hyperthyroidism cases, can predict patients with equivocal results or those that might be subclinical for further medical assessment and follow-up. A Wayne’s score can give the results of hyperthyroidism comparable to the results obtained by biochemical investigations. This tool is not in common use, but it is quite useful and should be put into broader usage.

## Introduction

Since the discovery of oil in Saudi Arabia, which positively affected its socioeconomic status, notable improvements have been seen across many sectors of the country. Healthcare is one of the sectors that has undergone remarkable advancements. The government has allocated large amounts of oil revenue to develop the healthcare system in the country. The healthcare organizations in Saudi Arabia deliver high-quality, up-to-date services that provide the best patient outcomes. Health awareness levels have increased in Saudi Arabian society due to the beneficial progress in the healthcare system [1]. Early discovery and prevention of diseases such as endocrine disorders are essential to reduce both prevalence and associated costs. Thyroid diseases are common endocrine disorders in Saudi Arabia and the rest of the Middle East but are usually misunderstood or misdiagnosed [2].

Hyperthyroidism is one of the endocrine disorders that occurs due to excessive production of the thyroid hormones, which are triiodothyronine and thyroxine, and characterized by low serum thyroid-stimulating hormone. Physical examination remains paramount to detect hyperthyroidism if a blood test is not available. Wayne’s index score is a clinical tool used to establish a clinical diagnosis for hyperthyroidism [3].

Hyperthyroidism is a hypermetabolic disorder that is difficult to diagnose because the symptoms can mimic many other diseases and could lead to an inaccurate diagnosis and mismanagement. If untreated, this can lead to significant mortality [4]. The exact cause of hyperthyroidism is not reliably defined; however, the most common cause of thyroid disorders is iodine deficiency [5]. Hyperthyroidism’s common symptoms are nervousness, intolerance of heat, weight loss despite appetite increase, and the body becoming warm and wet [6]. The main hyperthyroidism disorders are Graves’ disease, thyroid storm, toxic thyroid nodule, toxic nodular struma, and hashitoxicosis [2].

As the healthcare system leans toward limiting the use of many diagnostic tests, Wayne’s index has been useful in diagnosing hyperthyroidism. According to Naraintran et al., Sir Edward Wayne had implemented the index to find a primary result through a quantification score that will provide physicians with results to perform a referral [3]. Wayne’s index is a clinical diagnostic tool to enhance the accuracy of hyperthyroidism assessments. The score will be divided into three parts: the people who will get a score above 19 are considered to have toxic hyperthyroidism, a score from 11 to 19 is equivocal, and less than 11 is considered euthyroidism [7]. The present study aims to estimate the prevalence of suspected hyperthyroidism cases by using Wayne’s index score and the factors that increase the risk of suspected cases of hyperthyroidism.

## Materials and methods

The study was a cross-sectional analytic study performed in Jeddah (Kingdom of Saudi Arabia), conducted in October 2019 for five days during an awareness campaign organized by the College of Applied Medical Sciences of Jeddah University for the public in two shopping malls in Jeddah, Saudi Arabia. The campaign was held in the Red Sea and the Arab malls from 17 to 19 of October and 24 to 25 of October, respectively.

The inclusion criterion was for the participant to be older than age 16. Participants who had a history of any thyroid disorders were excluded from the study.

The study was conducted using a self-administered Arabic questionnaire. An endocrinology consultant validated the questionnaire. It consists of three parts to assess the prevalence of suspected hyperthyroidism cases in Jeddah by using Wayne’s scoring index. The first part was composed of questions regarding demographic data, the second part consisted of questions about thyroid disease symptoms, and the third part was related to thyroid gland examination.

The data were collected by physicians working in the medical centers of Jeddah University. They received training by one of the study investigators on each section of the questionnaire. Informed consent was obtained from the participants. Physicians sat with the participants and supervised them when they were filling out the first part of the questionnaire. Then, they asked the participants questions about the symptoms of thyroid diseases, according to Wayne’s index. After that, they obtained permission from the participants to do the thyroid examination according to Wayne’s index. The examination was done in private places, especially for women. Then, the scores of the participants were calculated. When the participants’ scores were equal or more than 11, physicians informed them that they had a risk of developing hyperthyroidism and advised them to go to the nearest primary healthcare center to have laboratory tests for hyperthyroidism confirmation and follow-up.

The sample size was calculated using OpenEpi, assuming the total targeted population is 3,000,000 individuals, and based on a previous study on the Saudi population that concluded the prevalence of hyperthyroidism was 2.8% [8]. The calculated sample size required was 346 individuals at an approximately 93.5% confidence limit.

The Research Ethics Committee approved this study at the College of Applied Medical Sciences of Jeddah University. Physicians informed all participants that all information would be kept confidential and will not be accessed except for scientific research.

IBM SPSS Statistics for Windows, Version 20.0. (IBM Corp., Armonk, NY) was used for data entry and analysis. The records were double-checked and entered by two researchers. Data were then analyzed using descriptive statistics. A Chi-square test was performed to identify the odds ratio, relative risk, positive predictive value, and negative predictive value between variables such as family history, chronic disease with the thyroid disease at a significance level of 0.05.

## Results

A total of 346 participants took part in the study. The age of the participants ranged from 16 to 56 years. The mean age of the participants was 32 years (SD = 9.6). More than half of the participants (n = 194, 56.1%) were women (152 men; 43.9%) and had a bachelor’s degree (n = 175, 50.6%; Table 1), and 75.7% (n = 262) have a family medical history free of thyroid disorders (Table 2).

**Table 1:** Distribution of sample study according to their sociodemographic data

		Frequency	Percent
Age	16 - 25 years	81	23.4%
	26 - 35 years	102	29.5%
	36 - 45 years	77	22.3%
	46 - 55 years	60	17.3%
	More than 56 years	26	7.5%
Gender	Men	152	43.9%
	Women	194	56.13%
Education	Primary	7	2.0%
	Intermediate	12	3.5%
	High school	91	26.3%
	Post	31	9.0%
	Diploma	24	6.9%
	Bachelor	175	50.6%
	Uneducated	6	1.7%

**Table 2:** Distribution of the study sample according to their family history of thyroid diseases

Have you ever been diagnosed a family member with thyroid diseases?	Frequency	Percent
Yes	84	24.3%
No	262	75.7%
Total	346	100.0%

According to Wayne’s scoring, more than two-thirds of the participants (n = 279, 80.6%) were euthyroid, 18.2% (n = 63) were equivocal, and only 1.2 % of the participants (n = 4) showed signs of hyperthyroidism (Table 3).

**Table 3:** Distribution of the study sample according to Wayne’s scoring

Thyroid function	Frequency	Percent
Euthyroid	279	80.6%
Equivocal	63	18.2%
Hyperthyroid	4	1.2%
Total	346	100.0%

The Chi-square test was used to test the association between Wayne’s scores and the participants’ sociodemographic characteristics. The results demonstrated that age, gender, level of education, and family history of chronic diseases, including thyroid disorders, were not significantly associated with hyperthyroidism (Tables 4-6).

**Table 4:** Association of Wayne’s score with age

Age		Wayne’s score		P-value
		Negative	Positive	
16 - 25 Years	Count	67	14	0.142
	% Within age	82.7%	17.3%	
26 - 35 Years	Count	75	27	
	% Within age	73.5%	26.5%	
36 - 45 Years	Count	62	15	
	% Within age	80.5%	19.5%	
46 - 55 Years	Count	54	6	
	% Within age	90.0%	10.0%	
More than 56 Years	Count	21	5	
	% Within age	80.8%	19.2%	

**Table 5:** Association of Wayne’s score with gender

Gender		Wayne’s score		P-value
		Negative	Positive	
Men	Count	123	29	0.905
	% Within gender	80.9%	19.1%	
Women	Count	156	38	
	% Within gender	80.4%	19.6%	

**Table 6:** Association of Wayne’s score with the level of education

Education		Wayne’s score		P-value
		Negative	Positive	
Primary	Count	5	2	0.599
	% Within education	71.4%	28.6%	
Intermediate	Count	10	2	
	% Within education	83.3%	16.7%	
High school	Count	68	23	
	% Within education	74.7%	25.3%	
Post	Count	25	6	
	% Within education	80.6%	19.4%	
Diploma	Count	20	4	
	% Within education	83.3%	16.7%	
Bachelor	Count	145	30	
	% Within education	82.9%	17.1%	
Uneducated	Count	6	0	

A Chi-square test was used to detect the association between Wayne’s scores and gender and chronic diseases (Table 7). The results showed that a history of having bronchial asthma was significantly associated with hyperthyroidism in men, while obesity was significantly associated with hyperthyroidism in women. However, Wayne’s scores were not significantly associated with gender, among other chronic diseases.

**Table 7:** Association of Wayne’s score with gender and chronic diseases

Gender		Male		Female	
Wayne’s score		Negative	Positive	Negative	Positive
Thyroid disorder	No	122	29	152	38
	Yes	1	0	4	0
P-value		0.626		0.319	
Diabetic	No	109	28	144	34
	Yes	14	1	12	4
P-value		0.198		0.569	
Blood pressure	No	111	27	143	35
	Yes	12	2	13	3
P-value		0.632		0.930	
Obesity	No	115	28	154	35
	Yes	8	1	2	3
P-value		0.531		0.021	
Heart disease	No	119	28	153	38
	Yes	4	1	3	0
P-value		0.957		0.389	
Asthma	No	123	28	154	38
	Yes	0	1	2	0
P-value		0.039		0.483	

## Discussion

According to the published medical records and to the best of our knowledge, this study is the first to address hyperthyroidism using Wayne’s score as a tool of assessment in the Kingdom of Saudi Arabia and other Arab countries. However, the published reports in the Kingdom used biochemical and other clinical methods [9,10]. Therefore, this study’s findings were compared to findings derived from biochemical testing due to the lack of reports of Wayne’s score. One study in India evaluated Wayne’s score and concluded that it had 86.9 % sensitivity and 96% specificity [3]. Another study from Nigeria compared Wayne’s scoring of participants to their biochemical testing of thyroid hormones, and they found a similar outcome [11]. Wayne’s score has high reliability, is easy, and a noninvasive tool for assessing hyperthyroidism, but unfortunately, it is rarely used. In this study, we found that 1.2% of the study sample conformed to the criteria of positive Wayne’s score, which means they have hyperthyroidism. This finding is consistent with the prevalence rate of hyperthyroidism reported in other studies. As an example, Hasanato et al. reported a 3% incidence of hyperthyroidism among women attending a hospital in Riyadh according to their collected history [12]. A study in Jordan reported the prevalence of hyperthyroidism as 1.8% in women and 2.27% in men [13]. A study from Sudan reported a prevalence of 3.4% of hyperthyroidism among pregnant women attending antenatal clinic in a hospital [14]. These reports, including our findings, were consistent with hyperthyroidism’s global prevalence, ranging from 0.2% to 1.3% [15]. The consistency of hyperthyroidism’s biochemical diagnosis with Wayne’s score supports its reliability as a practical noninvasive tool for diagnosing hyperthyroidism. According to Wayne’s score in this study, 18.2% of the participants were equivocal scores for hyperthyroidism. This finding cannot be ignored because those participants might be on the verge of hyperthyroidism or in a state of sub-clinical hyperthyroidism. This equivocal group needs further assessment and follow-up because they may develop overt hyperthyroidism in the future.

The association of age and gender in this study was insignificant for hyperthyroidism (P = 0.142). This finding was contradicted by other reports that linked hyperthyroidism significantly with increased age and female gender [16,17]. This could be attributed to the use of different methods for testing for hyperthyroidism. This study showed no significant difference between men and women in their scoring regarding hyperthyroidism. This finding did not agree with a common medical practice that thyroid dysfunctions, including hyperthyroidism, are more common in women. The most typical cause of hyperthyroidism is Graves’ disease, and autoimmune diseases are more common in women and therefore, hyperthyroidism is more common in them; this is generally accepted [12,16,17]. We found that education did not significantly differ in hyperthyroidism levels, which may reflect similar awareness among people of different educational levels. A study in Saudi Arabia reported that 57.32% of the sample lacked knowledge, and the rest had poor information about thyroid gland disorders [18]. In that report, age, gender, education level, and occupation had no significant effect on the degree of awareness about thyroid diseases. These findings were consistent with the outcome of this study [18].

Our study did not find a significant association between hyperthyroidism and history of thyroid disorders, diabetes mellitus, hypertension, and heart diseases. Bronchial asthma was found to be correlated with hyperthyroidism in men. Different studies reported similar findings, and hyperthyroidism reduces the chance of remission of bronchial asthma among men [19,20]. Obesity among women was found to be a significant factor for hyperthyroidism, according to this study. In the medical literature and clinical practice, hyperthyroidism is associated with weight loss due to a high basal metabolic rate that requires increased energy consumption. However, the patient’s appetite with hyperthyroid is increased, and sometimes the balance might be tipped toward weight gain due to excessive food intake. Such a finding was reported by Saeed et al. in a study from Iraq's northern region [21]. This indicates that obese people are also prone to develop hyperthyroidism; this is a fact that warrants notice by medical personnel. Moreover, the treatment of patients with hyperthyroidism can be followed by significant weight gain and even obesity, as reported [22,23].

## Conclusions

Wayne’s score is not in common use, but it is quite useful and should be put into broader usage. In our study, the prevalence of suspected hyperthyroidism cases in Jeddah city by using Wayne’s scoring was quite common.

Further studies require the measurement of thyroid function tests of the suspected cases and to evaluate the association between bronchial asthma and obesity with hyperthyroidism in men and women respectively.
